# Le mélanome malin de la fosse nasale

**Published:** 2012-07-11

**Authors:** Wafa Rekik, Aida Goucha, Mohamed Moncef Hamdane, Boutheina Debbabi, Ahmed EL May, Amor Gammoudi

**Affiliations:** 1 Service d’Immuno-histo-cytologie, Institut Salah Azaiez, Bab Saadoun, Tunis, Tunisie

**Keywords:** Mélanome, cavité nasale, anatomie pathologique, pronostic, Melanoma, Nasal cavity, pathology, prognosis

## Abstract

Le mélanome muqueux primitif de la fosse nasale est une tumeur maligne très agressive, rare, constituant 1% de l’ensemble des mélanomes. Sa symptomatologie est non spécifique et son diagnostic est anatomo-pathologique, appuyé par l’immunohistochimie. Son traitement est essentiellement chirurgical basé sur une résection large de la tumeur. Le pronostic est défavorable, caractérisé par des récidives et des métastases fréquentes et un taux de survie bas. Nous rapportons dans ce travail un nouveau cas de mélanome malin primitif de la fosse nasale, tout en précisant les caractéristiques de cette tumeur.

## Introduction

Le mélanome muqueux primitif de la fosse nasale est une tumeur maligne très agressive, décrite pour la première fois par Lucke en1869 [1, 2]. Il s’agit d’une entité rare, constituant entre 0,6 et 1% de l’ensemble des mélanomes [1, 3]. Nous rapportons dans ce travail un nouveau cas de mélanome malin primitif de la fosse nasale, tout en précisant les caractéristiques de cette tumeur.

## Patient et observation

**Figure 1:**
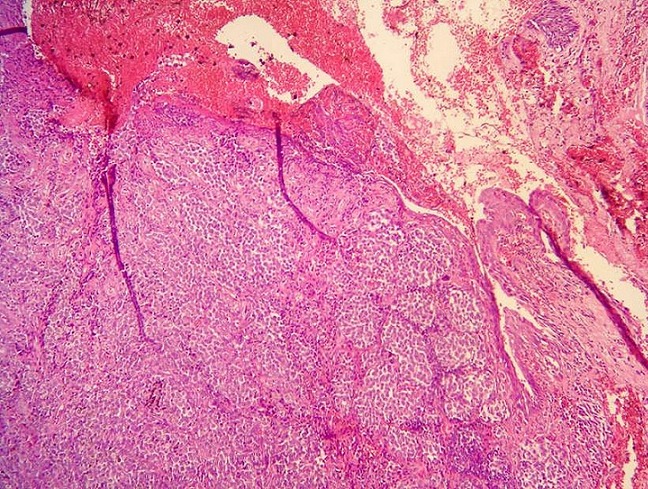
Prolifération tumorale d’architecture vaguement nodulaire (HEX40)

**Figure 2:**
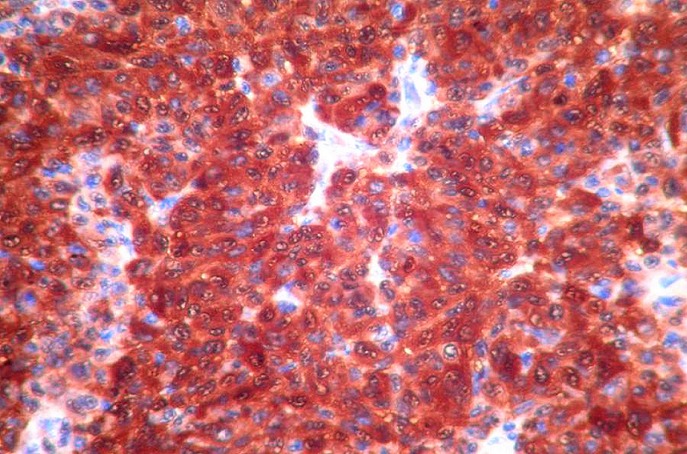
Immunohistochimie: positivité diffuse nucléaire et cytoplasmique à l’anticorps anti-protéine S100

**Figure 3:**
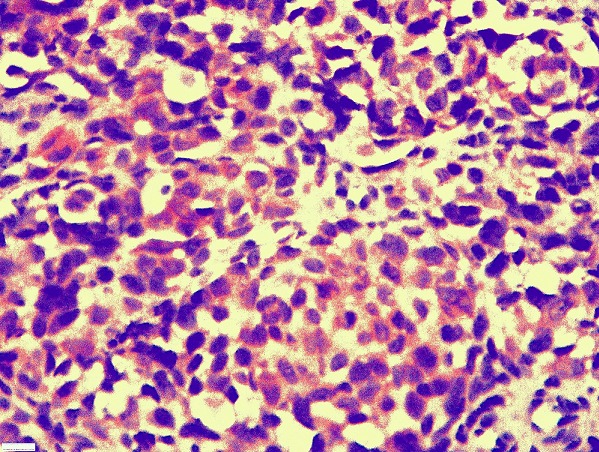
Immunohistochimie: positivité diffuse cytoplasmique à l’anticorps anti-HMB45

Une patiente âgée de 61 ans sans antécédents pathologiques particuliers était hospitalisée pour l’exploration d’une métastase ganglionnaire d’un mélanome. Cliniquement, la patiente était asymptomatique. Elle présentait des adénopathies sous digastrique et rétro spinale. L’examen clinique ainsi que les examens complémentaires n’avaient pas retrouvé le foyer primitif. L’évolution a été marquée par l’apparition 5 ans plus tard d’une obstruction nasale droite permanente, associée à une épistaxis et une rhinorrhée droite. La rhinoscopie a montré une formation bourgeonnante comblant la fosse nasale droite et arrivant jusqu’au vestibule narinaire droit. L’examen tomodensitométrique du massif facial a montré un comblement tissulaire de la fosse nasale droite et du sinus maxillaire droit sans lyse osseuse associée. Le cavum et l’oropharynx étaient libres. Une biopsie de la fosse nasale a été pratiquée. L’examen anatomopathologique a montré une prolifération tumorale maligne d’architecture vaguement nodulaire, au sein d’un stroma grêle fibro-inflammatoire (Figure 1). La prolifération était faite de cellules globuleuses au centre et fusiformes en périphérie. Ces cellules étaient de grande taille, atypiques, munies d’un nucléole proéminent et présentant une activité mitotique élevée. Aucun pigment mélanique n’a été retrouvé. Une étude immunohistichimique a montré une positivité franche des cellules tumorales aux anticorps anti-protéine S100 (Figure 2), anti-HMB45 (Figure 3), anti-Mélan A et anti-desmine. Ces aspects histologiques confrontés aux résultats immunohistochimiques étaient en faveur d’un mélanome achromique de la fosse nasale. La patiente a eu une exérèse chirurgicale complète de la tumeur avec une radiothérapie adjuvante.

## Discussion

Le mélanome est une tumeur maligne développée au dépend des mélanocytes, cellules d’origine neuroectodermique, retrouvées au niveau de la peau et des différentes muqueuses dérivant de l’ectoderme et moins fréquemment au niveau des muqueuses dérivant de l’endoderme, telles que les muqueuses nasale, nasopharyngée, laryngée, trachéobronchique et oesophagienne [4].

Le mélanome de la fosse nasale est une tumeur rare, représentant environ1% de l’ensemble des mélanomes [1, 3] et 8% des mélanomes cervico-faciaux [5]. Il touche les deux sexes de la même fréquence et survient dans 80% des cas après l’âge de 50 ans, avec un âge moyen de 65 ans [1–3]. Le site de prédilection est le septum nasal, notamment sa partie antéro-inférieure, suivi par la paroi externe (cornet inférieur, puis moyen) [1, 5].

Sur le plan clinique, la symptomatologie est non spécifique et la tumeur se manifeste par une obstruction nasale dans 50% des cas et par une épistaxis récidivante dans 20% des cas [1]. L’examen cutané cervico-facial et général permet d’éliminer un mélanome primitif cutané [1]. L’examen des aires ganglionnaires est indispensable bien que les adénopathies soient exceptionnelles [1]. La rhinoscopie montre une masse tumorale, pigmentée ou, dans un tiers des cas, achromique, avec parfois une mélanose de la muqueuse voisine [1]. L’endoscopie nasale permet de préciser la topographie de la tumeur, d’évaluer son extension vers le nasopharynx et de pratiquer des biopsies. Les prélèvements biopsiques doivent être multiples et avec une pince adaptée, en évitant de morceler le tissu tumoral [1].

Le diagnostic positif de mélanome de la fosse nasale ne peut être fait qu’après étude anatomopathologique. Macroscopiquement, la tumeur est souvent polyploïde ou nodulaire sessile, de couleur brune-noirâtre ou achromique-rosée, en fonction de la présence de pigment mélanique. Elle mesure généralement entre 2 et 3cm mais peut être plus volumineuse. Les ulcérations, les remaniements hémorragiques et la nécrose sont fréquents [3]. Histologiquement, la tumeur est faite de massifs ou de travées de cellules atypiques, à noyaux pléomorphes, avec une activité mitotique souvent élevée. Un pigment mélanique cytoplasmique est retrouvé dans 80% des tumeurs. L’aspect des cellules tumorales est variable. Souvent, elles sont fusiformes et/ou épithélioïdes, de grande taille, à cytoplame éosinophile abondant et à noyaux arrondis munis de nucléoles éosinophiles proéminents. Dans 30 à 40% des cas la prolifération tumorale est faite de petites cellules rondes, indifférenciées, ressemblant à celles d’un lymphome. Ailleurs, les cellules peuvent être plasmocytoïdes, rhabdoïdes, pléomorphes ou claires. Souvent un mélange de toutes ces formes cytologiques est retrouvé [3]. L’immunohistochimie constitue un outil diagnostique précieux, notamment dans les mélanomes achromiques. Elle montre une positivité des cellules tumorales pour les anticorps anti-vimentine, anti-protéine S100, anti-HMB45 et anti-Mélan A [1, 3].

Une fois le diagnostic établi, un bilan pré thérapeutique doit être fait comportant un scanner cervico-faciale incluant les fenêtres osseuses, une IRM pour préciser au mieux l’extension extra sinusienne, une radiologie du thorax, une échographie abdomino-pelvienne et une scintigraphie osseuse [1, 5].

Le traitement des mélanomes des fosses nasales est essentiellement chirurgical, avec exérèse large de la tumeur, assurant une marge de sécurité de 2 cm au minimum [1, 3]. L’apport de la radiothérapie et de la chimiothérapie est discutable en fonction des auteurs [1, 3]. L’évolution est souvent défavorable. Les récidives se voient dans 60 à 80% des cas. Le taux de survie à 5 ans varie de 10 à 47% [3, 5]. Les métastases sont fréquentes et se voient surtout au niveau des poumons, du cerveau et du foie [3]. Elles sont rares au moment du diagnostic mais sont rencontrées dans 40 à 50% des cas au cours de l’évolution. Les facteurs pronostiques cliniques sont la taille de la tumeur, les récidives et les métastases ganglionnaires et à distance. Les prédicteurs histologiques de mauvais pronostic sont l’extension de la tumeur dans les tissus profonds, la présence d’une composante indifférenciée (petites cellules ou cellules bizarres) supérieure à 25%, une architecture papillaire ou sarcomatoïde, la présence de nécrose et les emboles vasculaires [3].

## Conclusion

Le mélanome de la fosse nasale est une tumeur rare, agressive dont le pronostic ne peut être amélioré que par un diagnostic précoce, un bilan pré-thérapeutique, loco-régional et général, minutieux et un traitement rapide basé sur une résection chirurgicale large de la tumeur.
